# Utility of serum *Aspergillus*-galactomannan antigen to evaluate the risk of severe acute exacerbation in chronic obstructive pulmonary disease

**DOI:** 10.1371/journal.pone.0198479

**Published:** 2018-06-05

**Authors:** Katsuhiro Yoshimura, Yuzo Suzuki, Yusuke Inoue, Koji Nishimoto, Kazutaka Mori, Masato Karayama, Hironao Hozumi, Kazuki Furuhashi, Noriyuki Enomoto, Tomoyuki Fujisawa, Yutaro Nakamura, Naoki Inui, Koushi Yokomura, Shiro Imokawa, Takafumi Suda

**Affiliations:** 1 Second Division, Department of Internal Medicine, Hamamatsu University School of Medicine, Hamamatsu, Japan; 2 Department of Respiratory Medicine, Shizuoka City Shimizu Hospital, Shizuoka, Japan; 3 Department of Clinical Pharmacology and Therapeutics, Hamamatsu University School of Medicine, Hamamatsu, Japan; 4 Department of Respiratory Medicine, Respiratory Disease Center, Seirei Mikatahara General Hospital, Hamamatsu, Japan; 5 Department of Respiratory Medicine, Iwata City Hospital, Iwata, Japan; National and Kapodistrian University of Athens, SWITZERLAND

## Abstract

**Background:**

Recent studies have shown that the microbiome, namely *Aspergillus* species, play a previously unrecognized role in both stable and exacerbated chronic obstructive pulmonary disease (COPD). Galactomannan is a major component of the *Aspergillus* cell wall that has been widely used as a diagnostic marker.

**Objectives:**

To explore whether serum levels of *Aspergillus*-galactomannan antigen could be used to evaluate the risk of severe acute exacerbation of COPD (AE-COPD).

**Methods:**

We measured the *Aspergillus*-galactomannan antigen levels of 191 patients with stable COPD, and examined its clinical relevance including AE-COPD.

**Results:**

There were 77 (40.3%) patients who were positive for serum *Aspergillus*-galactomannan antigen (≥0.5). High *Aspergillus*-galactomannan antigen level (≥0.7) was associated with older age and presence of bronchiectasis and cysts on computed tomography images. Compared to patients with low *Aspergillus*-galactomannan antigen level (<0.7), patients with high *Aspergillus*-galactomannan antigen level had significantly higher incidence of severe AE-COPD (*P* = 0.0039, Gray’s test) and respiratory-related mortality (*P* = 0.0176, log-rank test). Multivariate analysis showed that high *Aspergillus*-galactomannan antigen level was independently associated with severe AE-COPD (hazard ratio, 2.162; 95% confidence interval, 1.267−3.692; *P* = 0.005).

**Conclusion:**

Serum *Aspergillus*-galactomannan antigen was detected in patients with COPD, and elevated serum *Aspergillus*-galactomannan antigen was associated with severe AE-COPD.

## Introduction

Chronic obstructive pulmonary disease (COPD) is a common, preventable, and treatable disease. However, COPD imposes a significant burden on patients and is now the fourth leading cause of death in the world [1]. Acute exacerbation of COPD (AE-COPD) causes an acute worsening of respiratory symptoms, reduces patients’ quality of life (QOL), and exacerbates airflow limitations, which further increase the risk of future events. Subsequently, AE-COPD can cause an accelerated decline of pulmonary function, leading to death [1–3]. To prevent and determine the risk of future exacerbation episodes, the Global Initiative for Chronic Obstructive Lung Disease (GOLD) developed the ABCD assessment tool beyond the simple spirometric grading system [1]. Current GOLD guidelines recommend treatment strategies based on ABCD-estimated risk of AE-COPD.

*Aspergillus* species are ubiquitous, saprophytic fungi commonly found in humid soil, water, and decaying organic materials. These pathogens cause several types of respiratory diseases, such as invasive pulmonary aspergillosis (IPA), chronic pulmonary aspergillosis (CPA), and allergic bronchopulmonary aspergillosis (ABPA) [4–6]. Besides these respiratory diseases, sensitization and/or colonization of the respiratory tracts are not rare, and occur occasionally in the lower respiratory tracts [3, 7]. Although the pathogenesis of sensitization and colonization is not fully understood, they are thought to influence the development and progression of chronic respiratory diseases [6, 8–11]. A close association between fungal species and pathogenicity is frequently found in asthma; specifically, *Aspergillus* sensitization and/or colonization are associated with aggravation of respiratory symptoms and accelerated impairment of lung function [6, 12–14]. Filamentous fungi (mainly *Aspergillus*) have been isolated from sputum of 49% of patients with COPD, and sensitization to *Aspergillus* species is associated with worse lung function [5]. These findings suggest that *Aspergillus* species could be involved in the progression and prognosis of COPD. However, there have been no studies on the relationship between *Aspergillus* species and AE-COPD.

It is often difficult to diagnose *Aspergillus* disease in the clinical setting. However, *Aspergillus*-galactomannan antigen can be easily measured in serum samples, and thus has been widely used as a complementary diagnostic marker for the diagnosis of *Aspergillus* disease [15]. In this study, we investigated whether serum levels of *Aspergillus*-galactomannan antigen could be used to evaluate the risk of severe AE-COPD.

## Materials and methods

### Study design

This was a retrospective observational study conducted at Iwata City Hospital and Seirei Mikatahara General Hospital in accordance with the ethical standards described in the Declaration of Helsinki. The study was approved by the ethics committee of each institute (No. 2015–905 and No. 15–33). The need for patient approval and/or informed consent was waived, because the study was based on reviews of the patients’ records. This trial was registered with the UMIN Clinical Trial Registry (000026907).

### Patient eligibility

This study enrolled 191 consecutive patients with stable COPD eligible for simultaneous evaluation of serum *Aspergillus*-galactomannan antigen and pulmonary function between January 2006 and July 2015 (**S1 Fig**). None of the patients had experienced AE-COPD for more than one month before the serum *Aspergillus*-galactomannan antigen and pulmonary function measurements. A diagnosis of COPD was made according to GOLD criteria [1]. COPD patients who had any of the following were excluded: (i) any advanced malignant disease; (ii) insufficient follow-up period (less than 1 month); and (iii) high-resolution computed tomography (HRCT) findings consistent with Aspergilosis including IPA, CPA, or ABPA, sucha as cavities and/or air-crescent sign, pleural thickening, central bronchiectasis, and mucus plugging with bronchoceles [4, 5, 16].

### Serum *Aspergillus*-galactomannan antigen level and pulmonary function test

Serum *Aspergillus* galactomannan antigen level was measured with a sandwich enzyme-linked immunosorbent assay (ELISA) (Platelia™ *Aspergillus*; Bio-Rad Laboratories, Redmond, WA) at SRL Inc. (Tokyo, Japan). In some patients, *Aspergillus* IgG antibody level and *Aspergillus* IgE antibody level (cut-off ≥RAST class 2, corresponding to >0.07 U_A_/ml) [17] was measured by the Ouchterlony method and the ImmunoCAP FEIA method, respectively, also at SRL Inc.

Pulmonary function tests (PFTs) were performed in accordance with American Thoracic Society guidelines [18] using a Chestac-8900 system (Chest, Tokyo, Japan). The severity of airflow limitation was categorized as per GOLD guidelines [1].

### Definitions and clinical characteristics of severe AE-COPD

Clinical and laboratory data were collected based on medical records at the time of measuring serum *Aspergillus*-galactomannan antigen and performing the PFTs. AE-COPD was diagnosed according to GOLD criteria [1]. Severe AE-COPD was also defined as an AE that required hospital admission [19]. The cumulative incidences of severe AE-COPD, respiratory-related mortality, and overal survival were calculated. Death due to respiratory diseases, such as respiratory failure, adult respiratory distress syndrome, pneumonia, and AE-COPD, was defined as respiratory-related mortality [20]. Cardiovascular diseases were defined as ischemic heart disease, congestive heart failure, coronary heart disease, and peripheral vascular disease [21]. Hypertension and diabetes were defined based on patient reports, or use of medication for hypertension and diabetes.

### Radiographic findings

Chest HRCT images were available for all patients. HRCT scans with a slice thickness of 1 mm were obtained using a 64-channel multidetector (Aquilion™; Toshiba Medical Systems, Otawara, Japan). The HRCT images were reviewed independently by two experienced pulmonologists (KY and YS) who were blinded to the clinical data. Bronchiectasis was defined as the lack of tapering of bronchi and identification of bronchi in the central two-thirds of the lung field [22]. A cyst was defined as a round parenchymal lesion with a well-defined thin wall (<2 mm) [23].

### Statistical analysis

Discrete variables are expressed as numbers (percentages) and continuous variables are expressed as medians (ranges). Categorical variables were analyzed using Fisher’s exact test. Continuous variables were analyzed using the Mann–Whitney U-test, and the Kruskal–Wallis test. ROC curve analysis was employed to set optimal cut-off values for predicting severe AE-COPD. Cumulative incidence of severe AE-COPD was calculated from the date of *Aspergillus*-galactomannan antigen measured and was estimated using the method of Fine and Gray [24]. Any death was considered as a competing risk in the analysis. Univariate and multivariate analyses were performed by Fine–Gray’s proportional hazards model. The Kaplan–Meier method with log-rank test and univariate models with Cox proportional hazards regression analyses were performed to analyze respiratory-related mortality. Propensity-score matching started with the smallest population (61 patients in the high serum *Aspergillus*-galactomannan antigen subgroup), which was matched 1:1 to the largest subgroup. Statistical analyses were performed using R software (version 3.2.0; The R Foundation for Statistical Computing, Vienna, Austria) [25]. *P* values of less than 0.05 were considered significant.

## Results

### Clinical characteristics of patients with stable COPD

The clinical characteristics are shown in **Table 1**. Median age was 73 years (range, 34–93 years), and 173 (90.6%) patients were male. Median pack-years was 55.0 (range, 2.5–230). Fifty-four (28.3%) patients had hypertension, 21 (11.0%) had diabetes, and 53 (27.7%) had cardiovascular disease. The proportions of patients in the GOLD classification stages according to airflow limitation severity were as follows: 25.7% in stage I, 45.5% in stage II, 21.5% in stage III, and 7.3% in stage IV. Radiographic findings showed emphysema and bronchiectasis in 77.5% and 13.6% of the patients, respectively. Most of the patients were treated with long-acting muscarinic antagonists and/or long-actingβagonists, whereas 65 (34.0%) patients were administered inhaled corticosteroids (ICS). Long-term oxygen therapy was administered in 19 patients (9.9%).

**Table 1:** Characteristics of patients with COPD according to serum *Aspergillus*-GM antigen status.

Characteristics	Total	Serum *Aspergillus*-GM antigen status		*P*—value
			Low (<0.7)		High (≥0.7)
	N = 191	N = 130	N = 61	
**Sex, male**	173 (90.6)	117 (90.0)	56 (91.8)	0.795
**Age, years**	73 (34–93)	71 (34–93)	74 (59–91)	0.007
**Smoking, pack years**	50.0 (2.5–230)	50.0 (2.5–230)	50.0 (2.5–200)	0.782
**BMI, kg/m**^**2**^	20.7 (11.4–30.7)	20.7 (11.4–28.7)	20.6 (13.7–30.7)	0.938
**Comorbidities**				
Hypertension	54 (28.3)	38 (29.2)	16 (26.2)	0.732
Diabetes	21 (11.0)	16 (12.3)	5 (8.2)	0.466
Cardiovascular disease	53 (27.7)	34 (26.2)	19 (31.1)	0.492
**Pulmonary function test**				
FVC, L	2.87 (1.05–5.55)	2.91 (1.05–5.55)	2.77 (1.10–4.44)	0.053
%FVC, %	91.1 (34.0–137.9)	91.8 (34.0–137.9)	87.9 (48.1–125.6)	0.081
FEV_1_ /FVC, %	59.0 (28.5–69.7)	59.0 (28.5–69.7)	59.1 (32.5–69.3)	0.582
FEV_1_, L	1.59 (0.31–3.62)	1.65 (0.31–3.62)	1.45 (0.46–2.56)	0.066
%FEV_1_, %	63.6 (13.1–117.6)	67.8 (13.1–117.6)	57.5 (24.5–101.7)	0.132
GOLD I	49 (25.7)	38 (29.2)	11 (18.0)	0.339
GOLD II	87 (45.5)	57 (43.8)	30 (49.2)	
GOLD III	41 (21.5)	25 (19.2)	16 (26.2)	
GOLD IV	14 (7.3)	10 (7.7)	4 (6.6)	
**Radiographic findings**				
Emphysema	148 (77.5)	104 (80.0)	44 (72.1)	0.266
Bronchiectasis	26 (13.6)	13 (10.0)	13 (21.3)	0.042
Cyst	17 (8.9)	7 (5.4)	10 (16.4)	0.026
**COPD managements**				
LAMA	108 (56.5)	74 (56.9)	34 (55.7)	0.877
LABA	113 (59.2)	74 (56.9)	39 (63.9)	0.430
ICS	65 (34.0)	41 (31.5)	24 (39.3)	0.327
LTOT	19 (9.9)	11 (8.5)	8 (13.1)	0.313

Variables are presented as N (%) or Median (range).

Abbreviations: COPD, chronic obstructive disease; GM, galactomannan; BMI, body mass index; FVC, forced vital capacity; FEV_1_, forced expiratory volume in 1 second; LAMA, long-acting muscarinic antagonists; LABA, long-acting β agonists; ICS, inhaled corticosteroid; LTOT, long-term oxygen therapy.

### Serum *Aspergillus*-galactomannan antigen is associated with COPD

**Fig 1:**
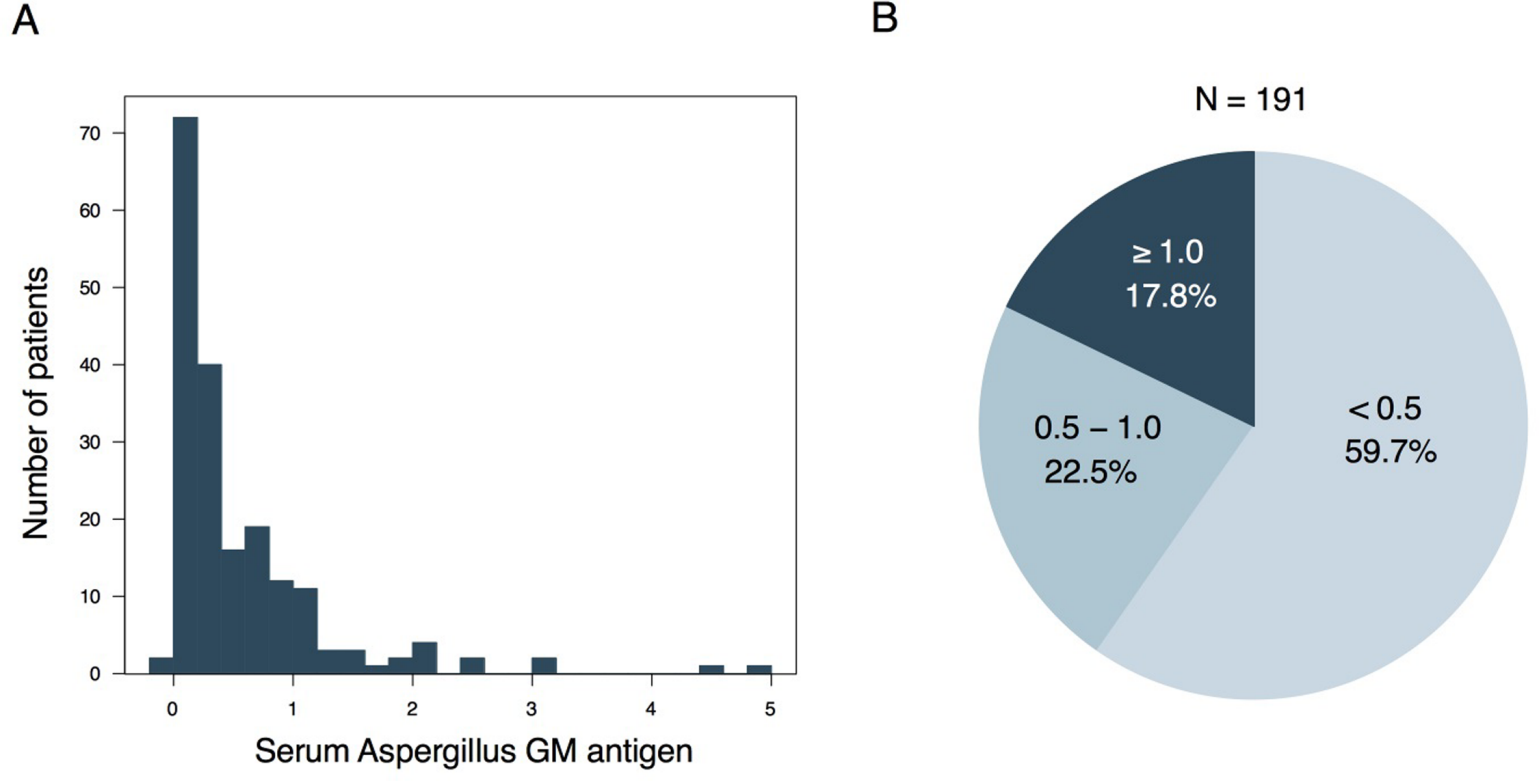
Measured serum *Aspergillus*-galactomannan antigen levels in stable patients with COPD. (A) Histogram of measured serum *Aspergillus*-galactomannan (GM) antigen in 191 stable patients with COPD. (B) Pie chart of the distribution of serum *Aspergillus*-GM antigen levels.

The serum levels of *Aspergillus*-galactomannan antigen in the enrolled patients are shown in **Fig 1**. Among the 191 patients with stable COPD, 114 (59.7%) had serum *Aspergillus* galactomannan antigen <0.5. The remaining 77 (40.3%) patients had serum *Aspergillus* galactomannan antigen >0.5, of which 34 (17.3%) patients had serum *Aspergillus* galactomannan antigen ≥1.0.

Next, we evaluated the association of serum *Aspergillus*-galactomannan antigen with severe AE-COPD. The cut-off value was set at 0.7 according to ROC analysis. Patients in the high *Aspergillus*-galactomannan antigen subgroup were significantly older (*P* = 0.007) and had significantly more bronchiectasis (*P* = 0.042) and cysts (*P* = 0.026) than patients in the low *Aspergillus*-galactomannan antigen subgroup (**Table 1)**. There were no significant differences in sex, body mass index (BMI), pulmonary function, comorbidities, and medication between high and low subgroups. Additionally, among the limited patients (N = 92, 48.2%) who were eligible for follow-up pulmonary function tests, there were no significant differences in lung function between the two subgroups **(S2 Fig)**.

### Serum *Aspergillus*-galactomannan antigen predicts severe AE-COPD

**Fig 2:**
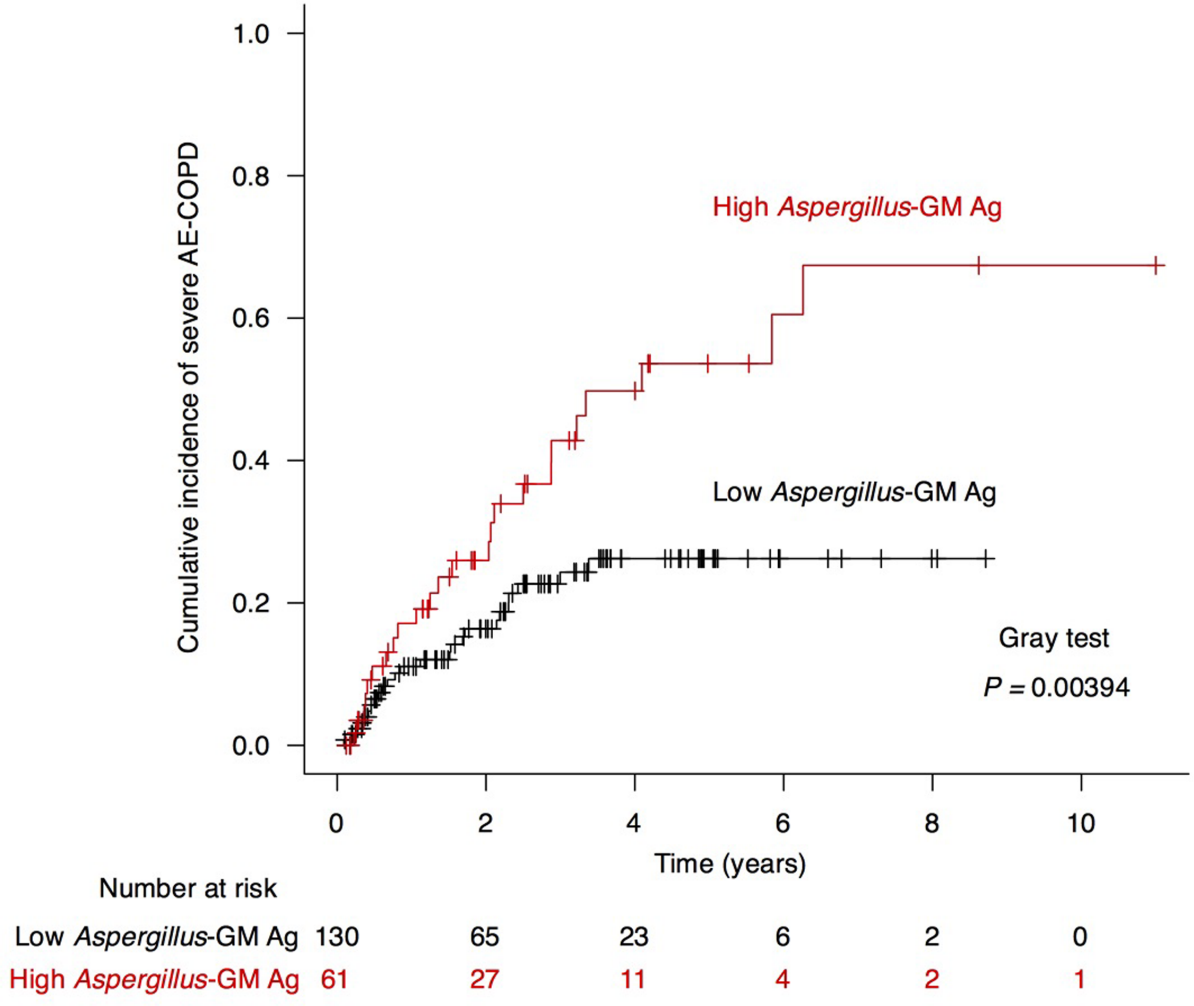
Cumulative incidence of severe AE-COPD according to serum *Aspergillus*-galactomannan antigen. The cumulative incidence of severe AE-COPD was determined using the Fine and Gray method. The red line represents the subgroup with high serum *Aspergillus*-galactomannan (GM) antigen level (≥0.7), and the black line represents the subgroup with low serum *Aspergillus*-GM antigen level (<0.7).

During the observation period (median, 32 months), 49 (25.7%) patients experienced severe AE-COPD. Using the aforementioned cut-off level of serum *Aspergillus*-galactomannan antigen, a significant higher incidence of severe AE-COPD was observed among patients in the high *Aspergillus*-galactomannan antigen subgroup (*P* = 0.004, Gray’s test; **Fig 2**). Univariate analysis with the Fine–Gray proportional hazard model showed that age, percentage of forced expiratory volume in 1 second (%FEV_1_), HRCT images, and high level of serum *Aspergillus* galactomannan antigen were significantly associated with severe AE-COPD (**Table 2**). After adjusting for age, sex, BMI, and other significant covariables, multivariate analysis showed that high *Aspergillus* galactomannan antigen level (hazard ratio [HR], 2.162; 95% confidence interval [CI], 1.267–3.692; *P* = 0.005) and %FEV_1_ (HR, 0.965; 95% CI, 0.950–0.980; *P* < 0.001) were independent predictive factors for severe AE-COPD.

**Table 2:** Prediction for severe AE-COPD with Fine-Gray proportional hazards model.

variable	Per unit for HR	Unadjusted HR	95% CI	*P—*value	Adjusted HR	95% CI	*P—*value
**Age**	10-years	1.552	0.941–2.558	0.085	1.046	0.989–1.107	0.120
**Sex**	Male/female	0.761	0.279–2.075	0.590	0.536	0.229–1.256	0.150
**BMI**	1-kg/m^2^	0.923	0.840–1.013	0.090	0.971	0.878–1.074	0.570
**Smoking**	10-pack years	1.005	0.998–1.013	0.170	-	-	-
**Comorbidities**					-	-	-
Hypertension	Yes/No	0.534	0.259–1.098	0.088			
Diabetes	Yes/No	0.853	0.348–2.090	0.730	-	-	-
Cardiovascular disease	Yes/No	1.283	0.708–2.325	0.410	-	-	-
Malignancy	Yes/No	0.807	0.460–1.414	0.450	-	-	-
**Pulmonary function tests**					-	-	-
%FVC	1-%	0.966	0.952–0.981	<0.001	-	-	-
%FEV_1_	1-%	0.965	0.953–0.978	<0.001	0.965	0.950–0.980	<0.001
**Radiographic findings**					-	-	-
Emphysema	Yes/No	1.463	0.700–3.057	0.310	-	-	-
Bronchiectasis	Yes/No	2.262	1.180–4.337	0.014	1.592	0.868–2.919	0.130
Cyst	Yes/No	1.498	0.654–3.435	0.340	-	-	-
**Serum *Aspergillus*** **galactomannan antigen**	High/Low	2.280	1.322–3.935	0.003	2.162	1.267–3.692	0.005

Abbreviations: AE, acute exacerbation; COPD, chronic obstructive disease; FVC, forced vital capacity; FEV_1_, forced expiratory volume in 1 second.

### High serum level of *Aspergillus*-galactomannan antigen is associated with poor COPD prognosis

**Fig 3:**
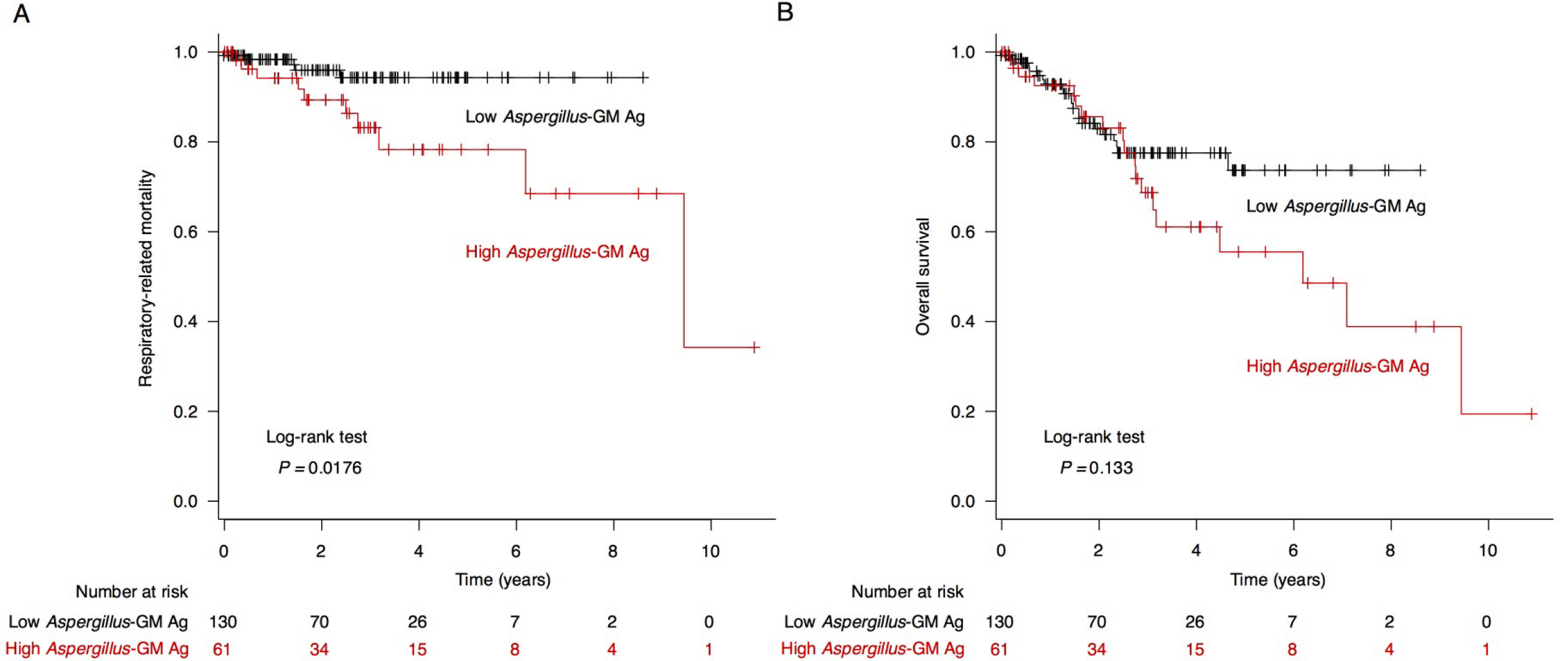
Survival curves according to serum *Aspergillus*-galactomannan antigen. Kaplan–Meier curves for (A) overall survival and (B) respiratory-related mortality according to measured serum *Aspergillus*-galactomannan (GM) antigen level. A significant difference in respiratory-related mortality was observed between high and low serum *Aspergillus*-GM antigen level subgroups.

Next, we evaluated the association of high serum *Aspergillus*-galactomannan antigen level with COPD prognosis. During the observation period, there were 41 (21.5%) deaths, including 15 (7.9%) respiratory-related deaths. We found that patients with high *Aspergillus*-galactomannan antigen had significantly worse respiratory-related mortality compared to patients with low *Aspergillus*-galactomannan antigen (*P* = 0.018, log-rank test; **Fig 3A**). Although there were no significant differences in all-cause mortality between the two subgroups, patients in the high *Aspergillus*-galactomannan antigen subgroup tended to show worse prognosis (*P* = 0.133, log-rank test; **Fig 3B**). Univariate analysis with Cox proportional hazard models also showed that high *Aspergillus*-galactomannan antigen level was significantly associated with higher respiratory-related mortality (HR, 3.493; 95% CI, 1.164–10.48; *P* = 0.026; **S1 Table**). Multivariate analysis of respiratory-related death was not performed because of the limited number of cases in our cohort[26].

### Propensity score-matched analysis confirms that serum *Aspergillus*-galactomannan antigen predicts severe AE-COPD

The patients in the high and low *Aspergillus*-galactomannan antigen subgroups showed some differences in demographic and clinical characteristics (**Table 1**). To account for these differences, we employed the propensity score-matching method to adjust age, sex, BMI, smoking status, pulmonary function, radiographic findings, comorbidities, and medication. We identified 61 patients with low *Aspergillus*-galactomannan antigen level whose characteristics were comparable to those of 61 patients with high *Aspergillus*-galactomannan antigen level (**S2 Table**). The cumulative incidences of severe AE-COPD were significantly higher in the high *Aspergillus*-galactomannan antigen subgroup than in the low *Aspergillus*-galactomannan antigen subgroup (*P* = 0.011, Gray’s test; **Panel A in S3 Fig**), and so were the incidences of respiratory-related mortality (*P* = 0.038, log-rank test; **Panel B in S3 Fig**), but not overall survival (**Panel C** in **S3 Fig**).

### Serum *Aspergillus* antibodies are not associated with serum *Aspergillus*-galactomannan antigen

Immunoglobulin E (IgE) or immunoglobulin G (IgG) of *Aspergillus* species is used to define sensitization [8, 12]. In this study, serum *Aspergillus* IgE and IgG antibody levels were measured in 67 and 29 patients, respectively. Eleven (16.4%) of 67 patients were positive for *Aspergillus* IgE antibody, and 11 (37.9%) of 29 patients were positive for *Aspergillus* IgG antibody. *Aspergillus* IgE and IgG antibody levels tended to associate, but not significantly so, with *Aspergillus*-galactomannan antigen levels (*P* = 0.158 and *P* = 0.108, respectively; **S4 Fig**). *Aspergillus* IgE and IgG antibodies were not associated with severe AE-COPD, respiratory-related mortality, or OS.

Sputum culture analyses were performed with 126 (66.0%) patients at the time of measuring *Aspergillus*-galactomannan antigen. *Aspergillus* species were isolated from only 2 (1.6%) patients, confirming *Aspergillus* colonization.

## Discussion

The present study measured serum *Aspergillus*-galactomannan antigen in patients with stable COPD and evaluated its association with severe AE-COPD. We found that 40% of patients with stable COPD were positive for serum *Aspergillus*-galactomannan antigen (≥0.5). The frequency of severe AE-COPD and respiratory-related mortality were significantly higher in patients with high level of serum *Aspergillus*-galactomannan antigen (≥0.7). Propensity score-matching analysis also revealed that high *Aspergillus*-galactomannan antigen level was an independent predictor of severe AE-COPD. Collectively, this study showed serum *Aspergillus*-galactomannan antigen as a novel surrogate marker that could potentially be used to evaluate the risk of severe AE-COPD.

*Aspergillus*-galactomannans are 35–100 kDa polysaccharides that form part of the cell wall of *Aspergillus* species [15]. Inhaled *Aspergillus* conidia bind to the airway surface via galactomannans, which subsequently activate the innate immune response [27, 28]. Detecting serum *Aspergillus*-galactomannan antigen is widely used for diagnosis of IPA, especially in immunocompromised hosts such as patients with hematological malignancy or a bone marrow transplant [16]. The sensitivity of serum *Aspergillus*-galactomannan antigen detection for the diagnosis of CPA is lower, at only 23% [29]. However, the sensitivity and specificity of *Aspergillus* galactomannan antigen in BAL are around 80% [30, 31]. Therefore, the detection of *Aspergillus*-galactomannan antigen in BAL, and not in serum, is recommended for the diagnosis of CPA [16].

In the present study, serum *Aspergillus*-galactomannan antigen was measured in patients with stable COPD, and surprisingly, 40% of them were positive for this antigen (≥0.5). Horie et al. reported that about 20% of patients with rheumatoid arthritis had nonspecific increased levels of serum *Aspergillus*-galactomannan antigen [32]. The incidences of false-positives have also been reported [15, 33, 34]. However, recent advances in the study of the microbiome have revealed a variety of microbial species in the airway that had not been previously identified using classical methods including mycotic cultures and its antigen tests, and showed that *Aspergillus* species are consistently found in the lower respiratory tract [35]. The present study excluded the spectrum of aspergillosis (obvious and/or suspected CPA, IPA, and ABPA by radiographic examination) and omitted possible cases of false-positives.

In the present study, the patients with high serum *Aspergillus*-galactomannan antigen level showed significantly more bronchiectasis and cysts, which might indicate moderate colonization or inflammation. Some of the patients were also positive for *Aspergillus*-IgG and/or IgE antibodies, suggesting previous sensitization or immune response to *Aspergillus*. Although the meaning of positive *Aspergillus*-galactomannan antigen in stable disease remains uncertain, it might reflect certain conditions such as moderate colonization or latent/inapparent infection rather than just existing antigens in the blood, which are independent of nonspecific elevations and/or false-positive results.

To manage COPD, it is essential to predict and prevent AE-COPD. AE-COPD causes mortality directly as well as indirectly by impairing QOL and worsening pulmonary function and symptoms [1]. Airflow limitation alone does not provide a good assessment of exacerbation risk [1, 2] and several other risk factors of AE-COPD have been proposed [1, 36, 37]. Among them, a history of prior exacerbation is the single best predictor of exacerbation [1, 2]. In fact, the GOLD guidelines suggest using a combination of history of exacerbation, history of hospitalization for exacerbation, and symptoms to assess the exacerbation risk [1]. In the present study, we found that high serum *Aspergillus*-galactomannan antigen level was an independent risk factor for severe AE-COPD. Furthermore, high serum *Aspergillus*-galactomannan antigen level was associated with respiratory-related mortality. Although the underlying mechanism is unknown, our findings highlight the potential of using serum *Aspergillus*-galactomannan antigen to evaluate the risk of severe AE-COPD.

*Aspergillus* species are known as a common and most important organism for patients with COPD. They cause critical ill including AE-COPD [38, 39], and reported that *Aspergillus* species were isolated from sputum in 16.6% of patients with severe AE-COPD [10]. Also a history of AE-COPD in the previous year was reported to be a main risk factor for isolation of Aspergillus species in the sputum samples [10]. Filamentous fungi and *Aspergillus* species were identified in 49% and 42% of sputum from patients with stable COPD, respectively, and *Aspergillus* cultures in COPD patients are related with neutrophilic inflammation, suggesting the presence of a host immune response against the *Aspergillus* organisms [9]. Additionally, galactomannan can activate the innate immune response by enhancing the activity of macrophages or dendritic cells via the recognition receptors DC-SING and pentraxin-3, subsequently leading to a cascade of innate immune response [28]. Galactomannan can induce persistent inflammation, which might increase the incidence of severe AE-COPD in patients with high level of *Aspergillus*-galactomannan antigen.

The clinical implications of *Aspergillus* infection in COPD patients are many-fold. For instance, the use of high-dose ICS and oral corticosteroids has been associated with *Aspergillus* colonization [9, 40]. On the other hand, isolation of *Aspergillus* species is not related with incidences of AE-COPD, decreased pulmonary function, and overall mortality [9, 10]. Additionally, there are no significant differences between *Aspergillus* colonization and serological tests for *Aspergillus* IgE and IgG antibodies [9]. Sensitization to *Aspergillus* is related with impaired lung function, but there are no reported relationships with AE-COPD or overall survival [8–10]. On the contrary, the present study showed an association between serum *Aspergillus*-galactomannan antigen and the incidences of severe AE-COPD, but not with airflow limitation or *Aspergillus* IgE and IgG antibodies. These distinct results might be explained by the unique characteristics of *Aspergillus* species, which exhibit potent hypersensitivity, innocuous, or virulent features that can coexist in individual patients. Therefore, our results must be interpreted while recognizing the heterogeneity of *Aspergillus* species.

Our study has some limitations. First, this study was a retrospective study. Although we have used propensity score-matching, there were potential confounding factors, and the selection of subjects could be biased. Therefore, further prospective studies are needed. Second, we only evaluated severe AE-COPD, and not mild–moderate AE-COPD, especially among outpatients. Additionally, as sensitivity of *Aspergillus*-galactomannan antigen in BAL were higher than the that in sera [30, 31], it would be interested to evaluate potential values of *Aspergillus*-galactomannan antigen levels in BAL for the predictive markers of AE-COPD. Finally, the causative pathogens of AE-COPD were not correctly identified. Therefore, the mechanisms underlying the association between *Aspergillus*-galactomannan antigen and severe AE-COPD should be revealed in the future work.

In conclusion, the present study demonstrated that serum *Aspergillus*-galactomannan antigen was detected in patients with COPD, and elevated serum *Aspergillus*-galactomannan antigen was associated with severe AE-COPD. These findings suggest serum *Aspergillus*-galactomannan antigen as a novel surrogate marker that could potentially be used to evaluate the risk of severe AE-COPD, and provide new grounds for understanding the interactions between the fungal microbiome and the pathogenesis of COPD.

## Supporting information

S1 Fig Flow diagram of patient selection. We screened patients with COPD between January 2006 and July 2015, and selected 277 eligible patients with COPD who underwent serum *Aspergillus*-galactomannan (GM) antigen examination. After applying the exclusion criteria, we enrolled 191 patients in the study. The patients were divided into low and high serum *Aspergillus*-GM antigen subgroups. Patient selection was based on 1:1 propensity-score matching.
(PDF)

S2 Fig Associations between serum *Aspergillus*-galactomannan antigen and pulmonary function decline. Absolute and percentage changes between baseline and secondary (more than 6 months) pulmonary function parameters, including FEV_1_, %FEV_1_, FVC, and %FVC. Each box plot indicates the median and interquartile range (top and bottom borders of the box). The whiskers above and below each box represent 1.5× of the interquartile range. There were no significant differences of worsening lung function between the high and low serum *Aspergillus*-galactomannan antigen subgroups. Abbreviations: FVC, forced vital capacity; FEV_1_, forced expiratory volume in 1 second.
(PDF)

S3 Fig Cumulative incidence of severe AE-COPD and prognoses for the matched cohort according to serum *Aspergillus*-galactomannan antigen. The cumulative incidence of severe AE-COPD (A) and Kaplan–Meier curves for respiratory-related mortality (B) and overall survival (C) according to measured serum *Aspergillus*-galactomannan antigen level. The red line represents the subgroup with high serum *Aspergillus* galactomannan antigen level (≥0.7), and the black line represents the subgroup with low serum *Aspergillus* galactomannan antigen level (<0.7). There were significant differences in the cumulative incidence of severe AE-COPD and respiratory-related mortality between high and low serum *Aspergillus*-galactomannan antigen level subgroups.
(PDF)

S4 Fig Associations between serum *Aspergillus*-galactomannan antigen and *Aspergillus*-related antibodies. The measured serum *Aspergillus*-galactomannan antigen level tended to be associated with positivity for *Aspergillus* IgE (A) and *Aspergillus* IgG (B). Each box plot indicates the median and interquartile range (top and bottom borders of the box). The whiskers above and below each box represent 1.5× of the interquartile range.
(PDF)

S1 Table Univariate analysis for predicting respiratory-related mortality with cox proportional hazards model. (DOCX)

S2 Table Characteristics of matched patients with COPD according to serum *Aspergillus*-galacomannan antigen status. (DOCX)
