# Supplementary figures and images for: Utility of serum Aspergillus-galactomannan antigen to evaluate the risk of severe acute exacerbation in chronic obstructive pulmonary disease
Source: PLoS One. 2018 Jun 5;13(6):e0198479. doi: 10.1371/journal.pone.0198479 (PMC5988315; doi:10.1371/journal.pone.0198479)

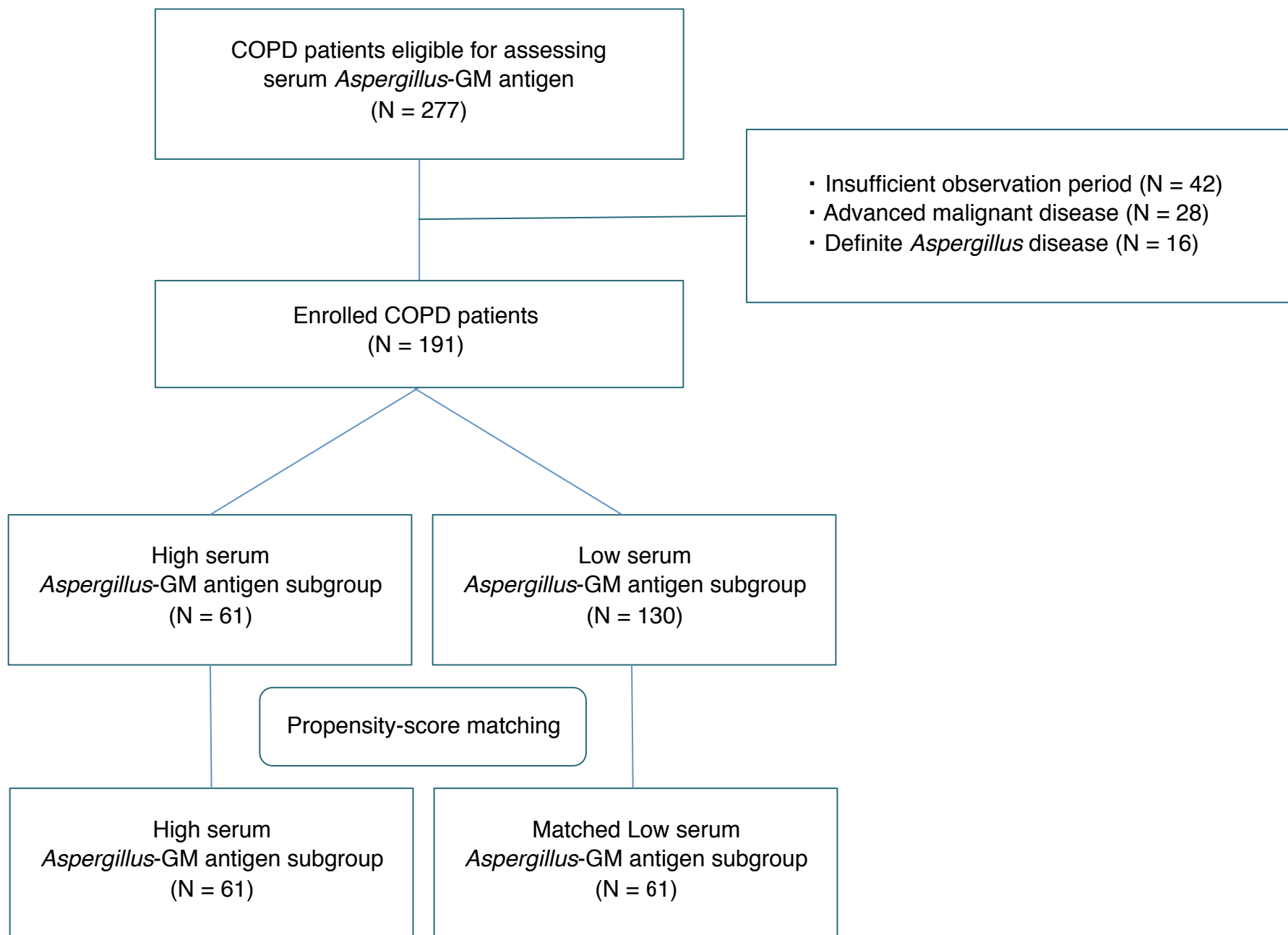

Supplement: S1 Fig — We screened patients with COPD between January 2006 and July 2015, and selected 277 eligible patients with COPD who underwent serum Aspergillus-galactomannan (GM) antigen examination. After applying the exclusion criteria, we enrolled 191 patients in the study. The patients were divided into low and high serum Aspergillus-GM antigen subgroups. Patient selection was based on 1:1 propensity-score matching. (PDF) [file pone.0198479.s001.pdf]

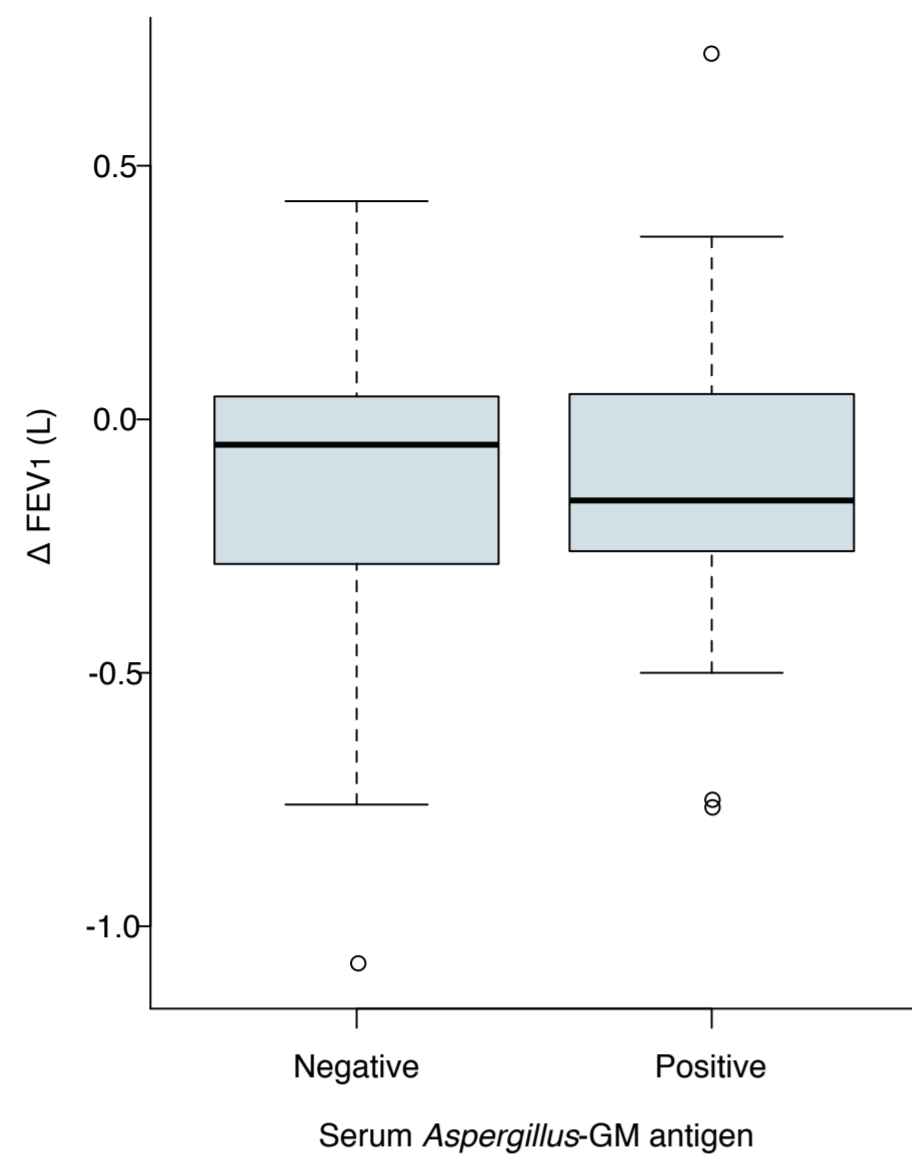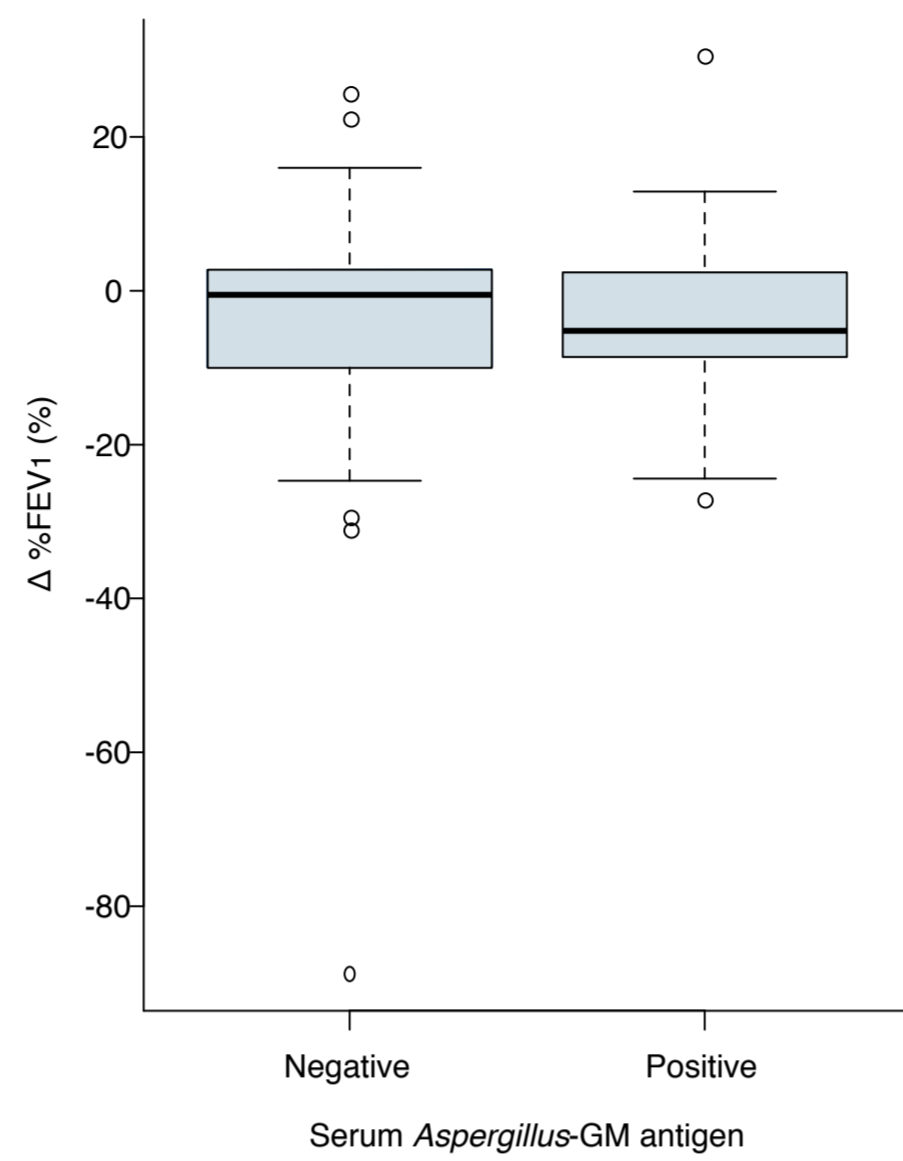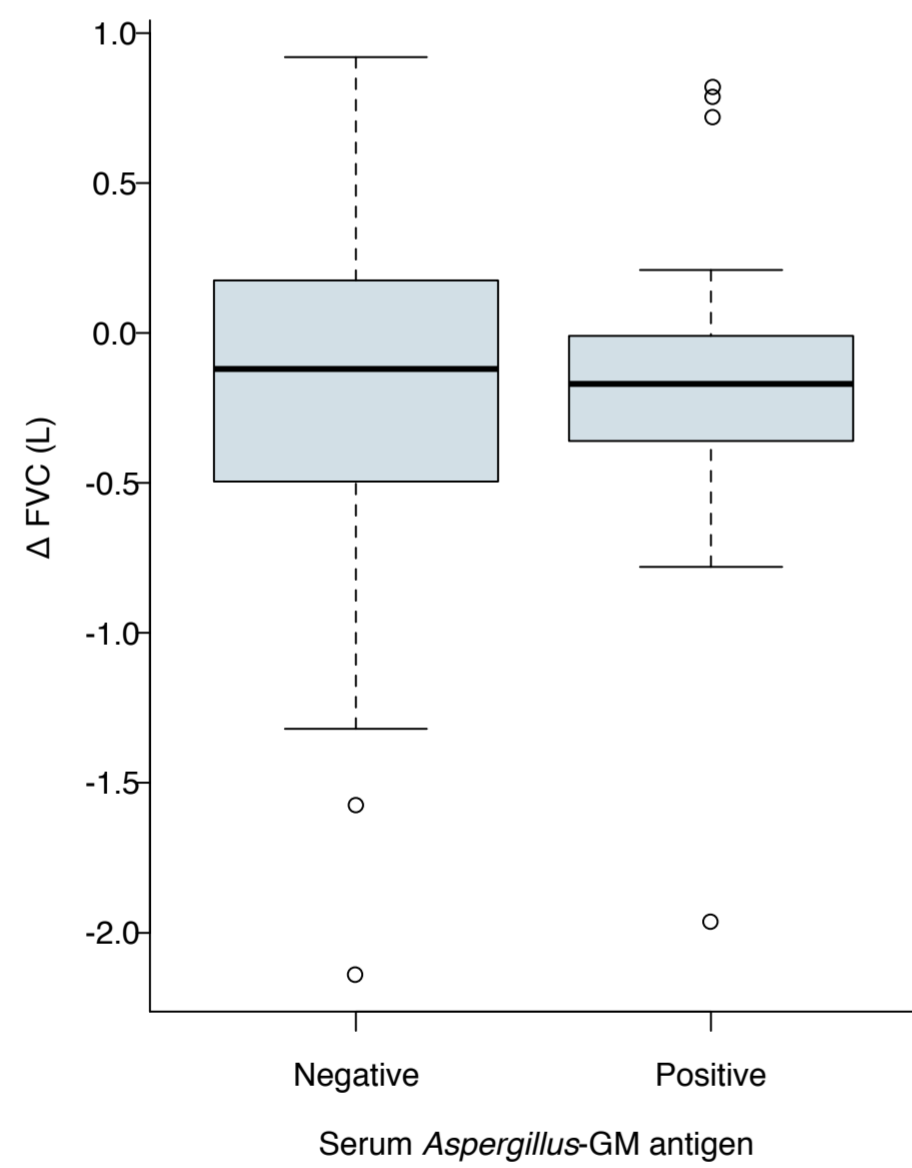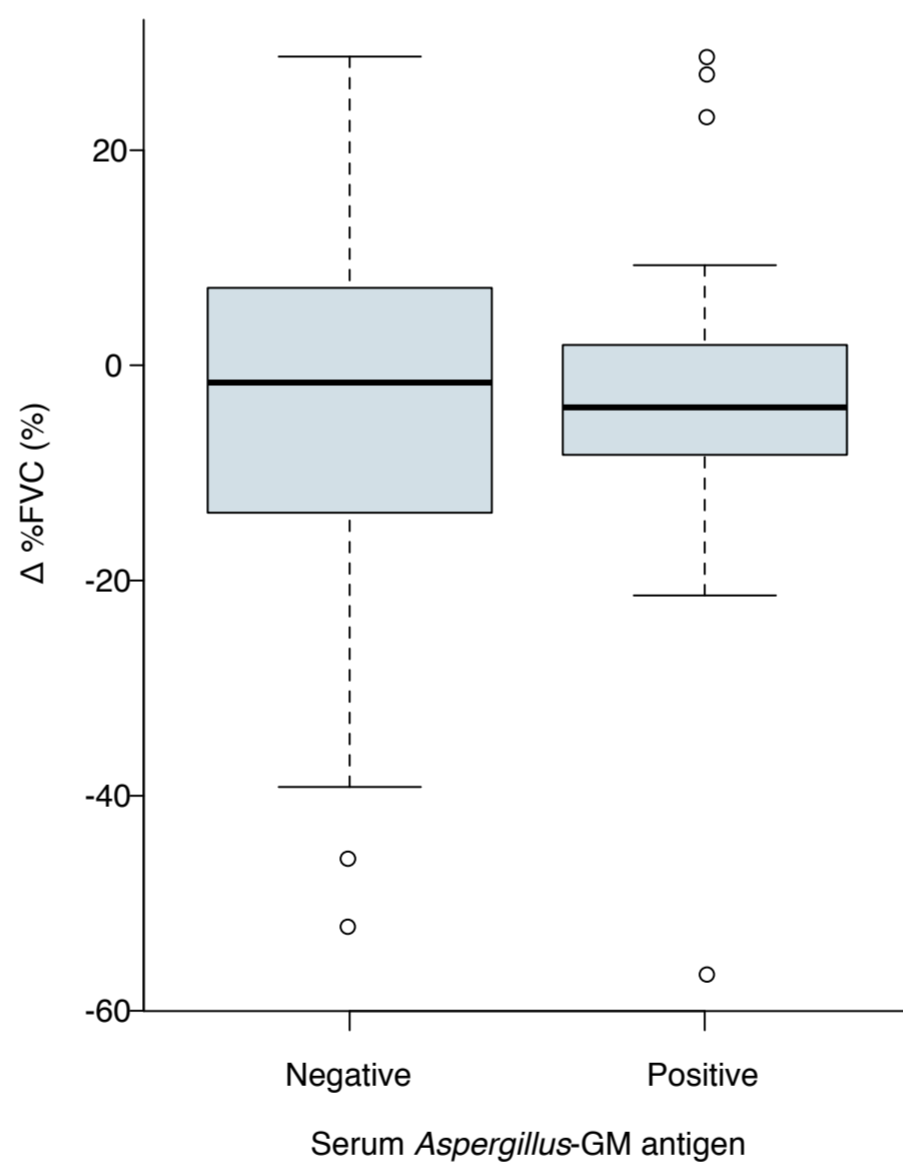

Supplement: S2 Fig — Absolute and percentage changes between baseline and secondary (more than 6 months) pulmonary function parameters, including FEV1, %FEV1, FVC, and %FVC. Each box plot indicates the median and interquartile range (top and bottom borders of the box). The whiskers above and below each box represent 1.5× of the interquartile range. There were no significant differences of worsening lung function between the high and low serum Aspergillus-galactomannan antigen subgroups. Abbreviations: FVC, forced vital capacity; FEV1, forced expiratory volume in 1 second. (PDF) [file pone.0198479.s002.pdf]

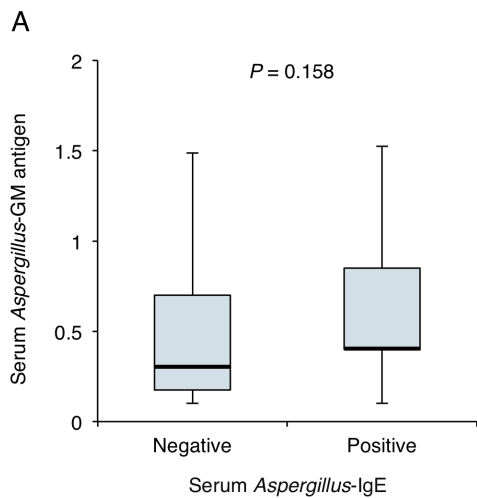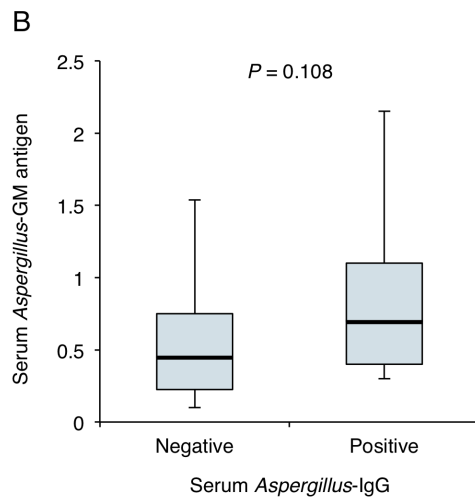

Supplement: S4 Fig — The measured serum Aspergillus-galactomannan antigen level tended to be associated with positivity for Aspergillus IgE (A) and Aspergillus IgG (B). Each box plot indicates the median and interquartile range (top and bottom borders of the box). The whiskers above and below each box represent 1.5× of the interquartile range. (PDF) [file pone.0198479.s004.pdf]
