# Supplementary material for: Utility of serum Aspergillus-galactomannan antigen to evaluate the risk of severe acute exacerbation in chronic obstructive pulmonary disease
Source: PLoS One. 2018 Jun 5;13(6):e0198479. doi: 10.1371/journal.pone.0198479 (PMC5988315; doi:10.1371/journal.pone.0198479)
Supplement: S3 Fig — The cumulative incidence of severe AE-COPD (A) and Kaplan–Meier curves for respiratory-related mortality (B) and overall survival (C) according to measured serum Aspergillus-galactomannan antigen level. The red line represents the subgroup with high serum Aspergillus galactomannan antigen level (≥0.7), and the black line represents the subgroup with low serum Aspergillus galactomannan antigen level (<0.7). There were significant differences in the cumulative incidence of severe AE-COPD and respiratory-related mortality between high and low serum Aspergillus-galactomannan antigen level subgroups. (PDF) [file pone.0198479.s003.pdf]

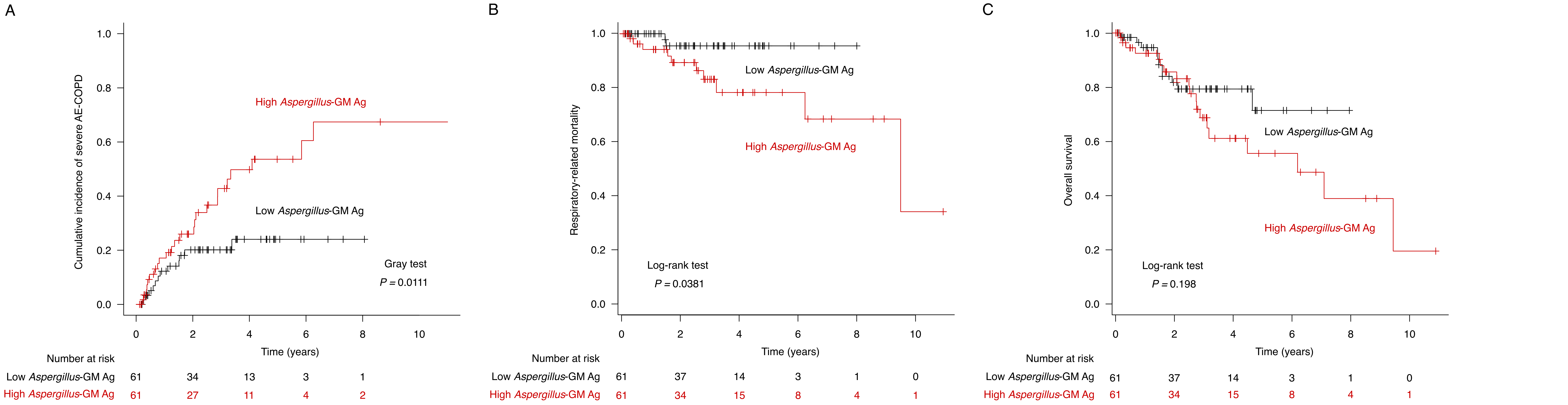
A
Cumulative incidence of severe AE-COPD
High Aspergillus-GM Ag
Low Aspergillus-GM Ag
Gray test
P = 0.0111
Time (years)
Number at risk
Low Aspergillus-GM Ag 61 34 13 3 1
High Aspergillus-GM Ag 61 27 11 4 2
B
Respiratory-related mortality
Low Aspergillus-GM Ag
High Aspergillus-GM Ag
Log-rank test
P = 0.0381
Time (years)
Number at risk
Low Aspergillus-GM Ag 61 37 14 3 1 0
High Aspergillus-GM Ag 61 34 15 8 4 1
C
Overall survival
Low Aspergillus-GM Ag
High Aspergillus-GM Ag
Log-rank test
P = 0.198
Time (years)
Number at risk
Low Aspergillus-GM Ag 61 37 14 3 1 0
High Aspergillus-GM Ag 61 34 15 8 4 1
