# Supplementary material for: Utility of serum Aspergillus-galactomannan antigen to evaluate the risk of severe acute exacerbation in chronic obstructive pulmonary disease
Source: PLoS One. 2018 Jun 5;13(6):e0198479. doi: 10.1371/journal.pone.0198479 (PMC5988315; doi:10.1371/journal.pone.0198479)
Supplement: S1 Table — (DOCX) [file pone.0198479.s005.docx]

**S1 Table. Univariate analysis for predicting respiratory-related mortality with Cox proportional hazards model.**

| variable | Per unit for HR | Unadjusted HR | 95% CI | *P -* value |
| --- | --- | --- | --- | --- |
| **Age** | 10-years | 2.693 | 1.276 - 5.682 | 0.009 |
| **Gender** | Male/female | 7.89E+07 | 0 - inf | 0.998 |
| **BMI** | 1-kg/m^2^ | 0.936 | 0.803 - 1.091 | 0.395 |
| **Smoking** | 10-pack years | 1.008 | 0.867 - 1.173 | 0.915 |
| **Comorbidities** |  |  |  |  |
| Hypertension | Yes/No | 1.257 | 0.426 - 3.712 | 0.679 |
| Diabetes | Yes/No | 0.755 | 0.098 - 5.818 | 0.787 |
| Cardiovascular disease | Yes/No | 2.337 | 0.839 - 6.512 | 0.104 |
| Malignancy | Yes/No | 2.89 | 0.920-9.085 | 0.069 |
| **Pulmonary function tests** |  |  |  |  |
| %FVC | 1-% | 0.984 | 0.958 - 1.011 | 0.239 |
| %FEV_1_ | 1-% | 0.99 | 0.966 - 1.015 | 0.431 |
| **Radiographic findings** |  |  |  |  |
| Emphysema | Yes/No | 0.998 | 0.314 - 3.174 | 0.997 |
| Bronchiectasis | Yes/No | 3.32 | 1.143 - 9.644 | 0.027 |
| Cyst | Yes/No | 2.218 | 0.616 - 7.979 | 0.223 |
| **Serum *Aspergillus galactomannan antigen*** | High/Low | 3.493 | 1.164 - 10.48 | 0.026 |
| Abbreviations: HR, hazard ratio; CI, confidence interval; FVC, forced vital capacity; FEV_1_, forced expiratory volume in 1 second. | | | | |
