# Supplementary material for: Utility of serum Aspergillus-galactomannan antigen to evaluate the risk of severe acute exacerbation in chronic obstructive pulmonary disease
Source: PLoS One. 2018 Jun 5;13(6):e0198479. doi: 10.1371/journal.pone.0198479 (PMC5988315; doi:10.1371/journal.pone.0198479)
Supplement: S2 Table — (DOCX) [file pone.0198479.s006.docx]

**S2 Table. Characteristics of matched patients with COPD according to serum Aspergillus-GM antigen status.**

| Characteristics | serum *Aspergillus*-GM antigen status | | *P* - value |
| --- | --- | --- | --- |
|  | Low (<0.7) | High (≥0.7) |  |
|  | N =61 | N =61 |  |
| **Sex, male** | 57 (93.4) | 56 (91.8) | 1.000 |
| **Age, years** | 73 (58-87) | 74 (59-91) | 0.183 |
| **Smoking, pack years** | 53 (16-129) | 50 (2.5-200) | 0.240 |
| **BMI, kg/m^2^** | 20.3 (15.2-28.7) | 20.6 (13.7-30.7) | 0.802 |
| **Comorbidities** |  |  |  |
| Hypertension | 16 (26.2) | 16 (26.2) | 1.000 |
| Diabetes | 5 (8.2) | 5 (8.2) | 1.000 |
| Cardiovascular disease | 14 (23.0) | 19 (31.1) | 0.689 |
| **Pulmonary function test** |  |  |  |
| FVC, L | 2.76 (1.25-4.51) | 2.77 (1.10-4.44) | 0.802 |
| %FVC, % | 86.6 (34.0-124.2) | 87.9 (48.1-125.6) | 0.943 |
| FEV_1_ /FVC, % | 54.8 (28.9-68.8) | 59.1 (32.5-69.3) | 0.488 |
| FEV_1_, L | 1.43 (0.67-2.48) | 1.45 (0.46-2.56) | 0.996 |
| %FEV_1_, % | 57.4 (23.1-103.2) | 57.5 (24.5-101.7) | 0.680 |
| GOLD Ι | 9 (14.8) | 11 (18.0) | 0.965 |
| GOLD ΙΙ | 33 (54.1) | 30 (49.2) |  |
| GOLD ΙΙΙ | 15 (24.6) | 16 (26.2) |  |
| GOLD ΙV | 4 (6.6) | 4 (6.6) |  |
| **Radiographic findings** |  |  |  |
| Emphysema | 50 (82.0) | 44 (72.1) | 0.282 |
| Bronchiectasis | 9 (14.8) | 13 (21.3) | 0.481 |
| Cyst | 5 (8.2) | 10 (16.4) | 0.270 |
| **COPD managements** |  |  |  |
| LAMA | 37 (60.7) | 34 (55.7) | 0.714 |
| LABA | 42 (68.9) | 39 (63.9) | 0.702 |
| ICS | 18 (29.5) | 24 (39.3) | 0.341 |
| LTOT | 7 (11.5) | 8 (13.1) | 1.000 |

Variables are presented as N (%) or Median (range).

Abbreviations: COPD, chronic obstructive disease; GM, galactomannan; BMI, body mass index; FVC, forced vital capacity; FEV1, forced expiratory volume in 1 second; LAMA, long-acting muscarinic antagonists; LABA, long-acting β agonists; ICS, inhaled corticosteroid; LTOT, long-term oxygen therapy.
